# The impact of equilibrium assumptions on tests of selection

**DOI:** 10.3389/fgene.2013.00235

**Published:** 2013-11-11

**Authors:** Jessica L. Crisci, Yu-Ping Poh, Shivani Mahajan, Jeffrey D. Jensen

**Affiliations:** ^1^Program in Bioinformatics and Integrative Biology, University of Massachusetts Medical SchoolWorcester, MA, USA; ^2^Swiss Institute of BioinformaticsLausanne, Switzerland; ^3^School of Life Sciences, École Polytechnique Fédérale de LausanneLausanne, Switzerland

**Keywords:** population genetics, statistical inference, positive selection, demography, simulation

## Abstract

With the increasing availability and quality of whole genome population data, various methodologies of population genetic inference are being utilized in order to identify and quantify recent population-level selective events. Though there has been a great proliferation of such methodology, the type-I and type-II error rates of many proposed statistics have not been well-described. Moreover, the performance of these statistics is often not evaluated for different biologically relevant scenarios (e.g., population size change, population structure), nor for the effect of differing data sizes (i.e., genomic vs. sub-genomic). The absence of the above information makes it difficult to evaluate newly available statistics relative to one another, and thus, difficult to choose the proper toolset for a given empirical analysis. Thus, we here describe and compare the performance of four widely used tests of selection: SweepFinder, SweeD, OmegaPlus, and iHS. In order to consider the above questions, we utilize simulated data spanning a variety of selection coefficients and beneficial mutation rates. We demonstrate that the LD-based OmegaPlus performs best in terms of power to reject the neutral model under both equilibrium and non-equilibrium conditions—an important result regarding the relative effectiveness of linkage disequilibrium relative to site frequency spectrum based statics. The results presented here ought to serve as a useful guide for future empirical studies, and provides a guide for statistical choice depending on the history of the population under consideration. Moreover, the parameter space investigated and the Type-I and Type-II error rates calculated, represent a natural benchmark by which future statistics may be assessed.

## Introduction

Population genetics seeks to characterize the forces that shape genomic variation, an endeavor that is often challenged by difficulties in unraveling the effects of selective and neutral processes. When positive selection acts on a new beneficial mutation, it will rise in frequency within a population over time, bringing nearby linked variation with it (Maynard Smith and Haigh, 1974). The pattern resulting from this process is referred to as a selective sweep, and can be observed in the site frequency spectrum (SFS) and the extent of linkage disequilibrium (LD) flanking the beneficial fixation [see reviews of Nielsen (2005); Crisci et al. (2012)]. Briefly, genetic variation within a swept region is expected to be reduced, and the SFS skewed toward an excess of both rare and high frequency derived mutations. The haplotype patterns surrounding the beneficial allele are expected to be significantly impacted (e.g., Stephan et al., 2006) as well—and it has thus, been suggested that a selective sweep may be identified by a characteristic haplotype pattern in which LD is increased in regions flanking a recent beneficial fixation, but reduced across the site of fixation (Jensen et al., 2007; Pavlidis et al., 2010).

Demographic forces also affect genetic variation and haplotype structure. For instance, spontaneous changes in population size can create longer haplotypes that may strongly resemble patterns expected after a selective sweep (Pavlidis et al., 2010). Additionally, as demonstrated by Barton (1998), the expected coalescent trees generated by a bottleneck may indeed be identical to those generated by selection, and simulation studies have demonstrated that tests of selection are prone to extremely high false positive rates under certain bottleneck models (e.g., Jensen et al., 2005; Thornton and Jensen, 2007).

Numerous methods for estimating selection and demography have been developed to deal with these challenges [for review see Thornton et al. (2007); Crisci et al. (2012)]. Many tests of selection have taken an outlier-based approach– thus, a statistic is computed across an entire dataset and a top fraction of values are considered selection candidates. Alternatively, a neutral model is simulated to match a sub-genomic region of interest and selective sweeps are identified based on outlier values of this neutral distribution. One limitation of this approach is the assumption of an equilibrium neutral background, with deviations being interpreted as evidence of non-neutrality (rather than non-equilibrium). While it has been proposed to first fit a demographic model in order to increase power to detect selective sweeps (e.g., Williamson et al., 2005; Keightley and Eyre-Walker, 2007), the demographic estimators themselves assume neutrality—and thus, the demographic fitting may account for much of the pattern in the data owing to selection.

We here focus on identifying selection in simulated recurrent hitchhiking (RHH) and single hitchhiking (SHH) datasets using four commonly used selection estimators: SweepFinder (Nielsen et al., 2005), SweeD (Pavlidis et al., 2013), OmegaPlus (Alachiotis et al., 2012), and iHS (Voight et al., 2006). We consider equilibrium and non-equilibrium neutral and selection models. Our intent is to characterize the demographic parameter space for which neutral and selective models may and may not be differentiated. Further, given the increasing number of proposed statistics in this area, we would like to emphasize the importance of proper power testing—and we here seek to describe performance across equivalent models. We hope that the statistical testing presented here, and the simulation panel assembled, may serve as a template against which future statistics may be evaluated allowing for a direct comparison with previously proposed methodology.

For our considered models, we find that the performance of the standard implementation of SweepFinder has very few rejections of neutrality under even equilibrium models with moderately strong selection (*2Ns* = 1000). SweeD had slightly improved performance, but mainly achieved a reduced sensitivity to SNP density owing to the inclusion of monomorphic sites. OmegaPlus was found to have the most power to detect selection, but remains prone to high false-positive rates under certain neutral non-equilibrium models. Finally, while iHS performs well under equilibrium conditions, it is unable to distinguish selective effects from those of a variety of population bottlenecks. Thus, in addition to serving as a benchmark for future studies, these results highlight the need for continued methodological development in this area, and emphasize the relative merits of LD relative to SFS based approaches.

## Methods

### Simulation parameters

Recurrent hitchhiking models (i.e., selective sweeps defined to occur at a specific rate) were simulated using sfs_code (Hernandez, 2008), a forward simulation program that can simulate both selection and demography simultaneously. SHH models (i.e., a single selective sweep occurs at a specified time) were simulated using msms, which can also model both selection and demography (Ewing and Hermisson, 2010). For both sets of models a single locus of 50 Kb was simulated using human-like parameters for population size *N* = 10,000, mutation rate (θ = 0.001/site), and recombination rate (ρ = 0.001/site). For each set of parameters, 1000 simulations were performed with 40 haplotypes sampled.

Selection parameters were set as follows: for SHH events, the selected allele was located in the center of the locus with 2*Ns* = 1000, 100, and 10 for homozygous alleles, and 500, 50, and 5, respectively, for heterozygous alleles. For RHH, selection occurs on a new mutation with a specified probability (=0.0002, 0.01, 0.1, or 0.25). Our models encompass equilibrium neutral, equilibrium selection, non-equilibrium neutral, and non-equilibrium selection—with bottlenecks ranging in severity from 25 to 99% size reduction and ranging in recovery time from 0.5 to 0.23 2N generations. A complete list of the parameters of mixture models can be found in each Table.

### Comparison of the different selection statistics

We evaluate selection statistics based on either the SFS (SweepFinder, SweeD) or patterns in LD (OmegaPlus, iHS) to identify regions that contain a selective sweep. These statistics were chosen because of their widespread use in population genetics, and for the public accessibility of their code.

SweepFinder uses information from the SFS to determine the probability of observing an allele at a given frequency and distance from a beneficial mutation (Nielsen et al., 2005, http://people.binf.ku.dk/rasmus/webpage/sf.html). This method is based on the similar framework of Kim and Stephan (2002), but the null SFS is determined from the background SFS rather than a strictly equilibrium neutral model. This approach has been argued to make the test more robust to demographic history and variation in mutation rate. SweepFinder is designed to detect completed sweeps in both subgenomic, and genomic datasets.

SweeD is a computationally improved version of SweepFinder that is capable of analyzing much larger datasets (thousands of sequences vs. hundreds for SweepFinder) in a cluster-computing environment (Pavlidis et al., 2013, http://sco.h-its.org/exelixis/software.html). The user can also optionally specify the use of monomorphic sites [explored in Pavlidis et al. (2010)], and can input parameters for an explicit demographic model to be used as the neutral SFS. SweepFinder requires a sufficiently SNP dense region in order to allow for accurate estimation, and the inclusion of a fraction of monomorphic sites evens out the SNP density as well as preserves the signature of low diversity in regions of depleted genetic variation (Pavlidis et al., 2010). Performance was evaluated with and without monomorphic sites.

OmegaPlus is a sliding-widow implementation of Kim and Nielsen's (2004) ω_MAX_ statistic that uses patterns of LD to identify selective sweeps (Pavlidis et al., 2013, http://sco.h-its.org/exelixis/software.html). It scans for windows of SNPs where there is increased LD flanking the fixation, and reduced LD across the fixation. Like SweeD, OmegaPlus is a high performance statistic capable of analyzing very large datasets.

Finally, we evaluated iHS as a second LD-based selection estimator (Voight et al., 2006, http://coruscant.itmat.upenn.edu/software.html). This is based on the EHH statistic, which measures the decay of LD from an individual SNP (Sabeti et al., 2002). Longer haplotypes will be observed when a SNP rises faster in frequency than would be expected under neutral conditions. iHS additionally looks at the LD decay of both the derived and ancestral state of each SNP, calculates EHH for both alleles, and then integrates the area between the two curves; the notion being that this area will be larger for a selected allele vs. a neutral allele. Because of the normalization step required for raw iHS scores, a large SNP dataset is necessary.

### Determining significance, and the effects of misspecification of the null

To determine the significance thresholds for SweepFinder, SweeD, and OmegaPlus, we simulated a range of neutral models in ms (Hudson, 2002) using the –s option to fix the number of segregating sites. After performing each test on this neutral set of models we determined the maximum value for each of 1000 iterations and used the 95th percentile as the cutoff value. The empirical models were then binned according to their average number of segregating sites and the 95th percentile value was used for each bin as a cutoff for significant test values. (i.e., *p* = 0.05).

**Figure 1:**
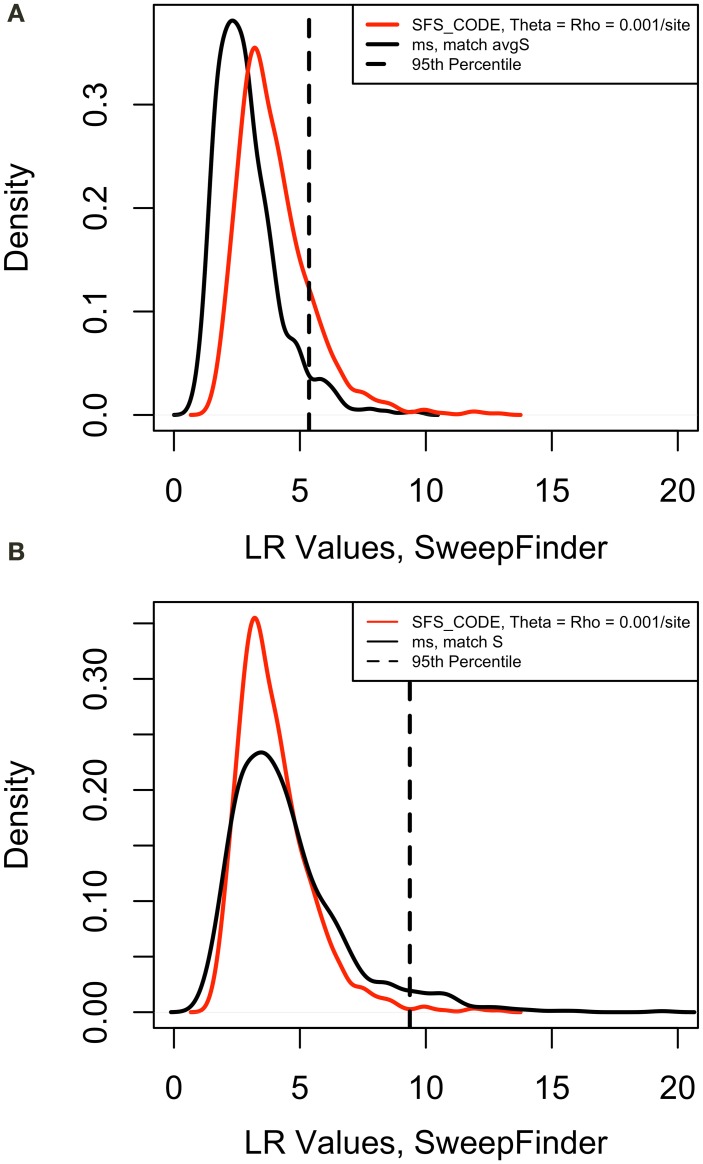
**Correction of False Positive Rate for Neutral Models.** Density plots for maximum likelihood ratio values for 1000 iteration of a neutral model. For sfs_code, theta = rho = 0.001 per site (red line). The same model was simulated in ms using the –s option to match the average number of segregating sites for the sfs_code model. The 95th percentile is for the ms model. **(A)** Sample size = 40, false positive rate = 0.15. **(B)** Sample size = 20, false positive rate = 0.01.

Next we verified that the distribution of test values in the cutoff models appropriately matched the values in the equilibrium neutral models simulated with sfscode. We observed that the distribution of values in the sfscode models were a poor match for the values obtained by running SweepFinder on the neutral models simulated in ms (Figure 1A). However, sfscode samples 2 haplotypes from 20 individuals (producing a sample size of 40), while ms samples 40 haplotypes from a diploid population (from separate individuals). Thus, a sample size correction is necessary for proper comparison (Figure 1B).

### Determining threshold for significant sweeps in iHS

The statistic iHS computes a test score for each SNP within a locus, whereas all previously mentioned statistics compute a test value at specific points across a user-specified grid. Since iHS requires a normalization step to control for SNPs at different frequencies, we followed a slightly different procedure to determine significance values for this test. Raw iHS scores were normalized according to the method described in Voight et al. (2006). Briefly, all SNPs across each dataset were binned according to frequency. The mean and standard deviation of each bin was calculated, and these values were used to normalize raw iHS scores in the following way: for each SNP, the mean of the corresponding bin is subtracted from the raw iHS score and this result is divided by the standard deviation. This produces iHS values with a mean of approximately 0 and variance 1 for each frequency such that all SNPs can be compared directly (Voight et al., 2006).

With iHS, extreme negative values indicate a derived allele on a long haplotype (potentially indicative of a selective sweep), and extreme positive values belong to a long ancestral haplotype. For this reason, the 1st percentiles were used to determine the significant values for the entire dataset.

## Results and discussion

### SFS-based statistics perform poorly under recurrent hitchhiking models

For equilibrium neutral models our initial false positive rates for SweepFinder approached 0.30. After correcting for the sample size as described above, the false positive rates were lowered to below 0.05, which is equivalent to a *p*-value of 0.05 (Table 1). However, this correction has the unfortunate property of lowering the rejection rate of SweepFinder for equilibrium selection as well. For 2*Ns* ranging from 10 to 1000, the true positive rate for SweepFinder and SweeD is also under 0.05 (i.e., the same rejection rate as neutral models; Table 2). OmegaPlus is the only statistic that has power to reject neutrality as the strength of selection is increased, with a true positive rate as high as 0.44. When the probability that a new mutation is affected by selection is increased, this reduces the rejection rate of OmegaPlus, which is consistent with fewer rejections at a lower SNP density (Table 1), and consistent with the poor performance of this LD-based approach under RHH models (Jensen et al., 2007).

**Table 1:**
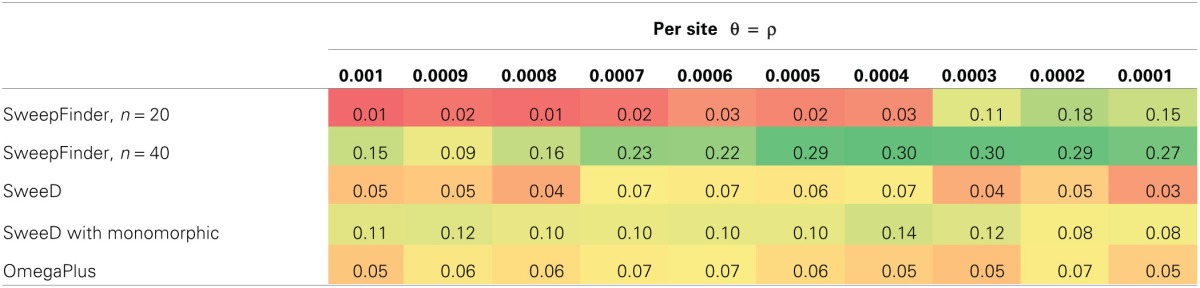
**False positive rate for equilibrium neutral models**.

*θ = ρ = 0.001/site. Corrected FPR is shown in top row (n = 20)*.

**Table 2:**
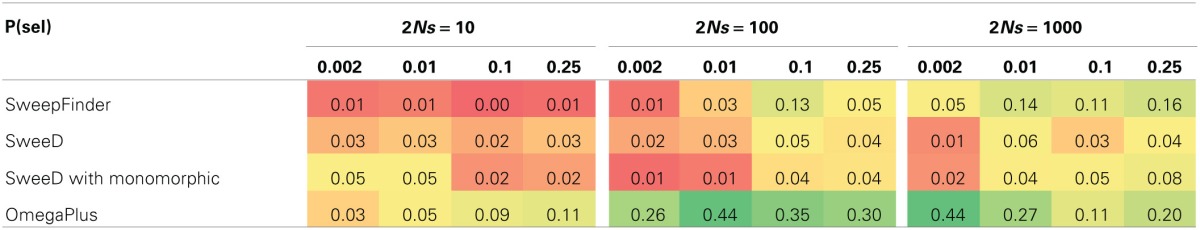
**True positive rate for equilibrium RHH models**.

*θ = ρ = 0.001/site*.

Our bottleneck models consist of a severity in reduction ranging from 25 to 99%, and duration ranging from 1000 to 4000 generations. Since sfscode is a forward simulator (and the reduction in population size begins at time 0), a longer duration is equivalent to a more recent recovery, whereas a shorter duration corresponds to an older bottleneck. In neutral bottleneck models, SweepFinder and SweeD have low power to reject for all parameter combinations (Table 3). OmegaPlus has a low false positive rate when the population size reduction is small, but for a 99% reduction, the rate of rejection is the same as for equilibrium selection models—suggesting an inability to distinguish these two scenarios. This is true for all duration times but is more pronounced as the recovery time decreases, with a false positive as high as 0.91 for a 99% reduction in population size that recovered only 0.05 2N generations ago (Table 3). Thus, severe population size reductions can mimic this pattern of LD normally attributed to selective sweeps, consistent with previous results (Pavlidis et al., 2010).

**Table 3:**
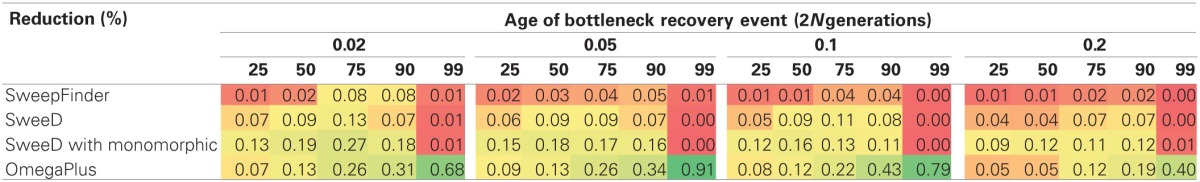
**False positive rate for neutral bottleneck models (sfscode)**.

*θ = ρ = 0.001/site. Duration for each model is 0.25 2N generations. All bottlenecks occur at time 0 with complete recovery (back to N = 10,000) at time 0.25—age of bottleneck recovery event*.

When a bottleneck is combined with strong selection (2*Ns* = 1000), SweepFinder shows a slightly improved propensity to reject the neutral model (Table 4), but power never exceeds 20%. OmegaPlus has a higher rejection rate at 2*Ns* = 100 vs. 1000, which is also likely due to the extreme reduction in genetic variation caused by combining strong selection with a bottleneck (Table 4). For non-equilibrium selection models the rate of rejection for OmegaPlus is within the same range as equilibrium selection models, which suggests that it is not capable of distinguishing selection from a bottleneck when both factors have impacted patterns of variation.

**Table 4:**
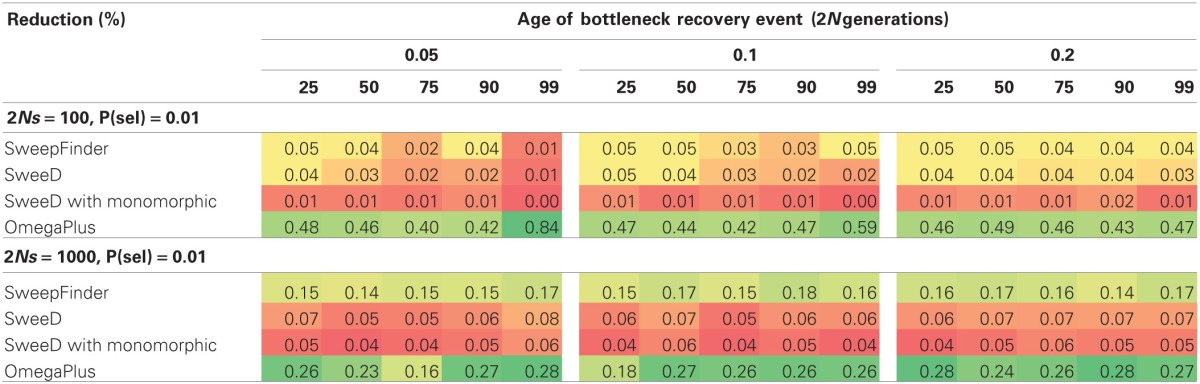
**True positive rate for joint RHH-bottleneck models**.

*θ = ρ = 0.001/site. Duration for each model is 0.25 2N generations. All bottlenecks occur at time 0 with complete recovery (back to N = 10,000) at time 0.25—age of bottleneck recovery event*.

### Single hitchhiking models

We included SHH models specifically to satisfy the sweep conditions for which SweepFinder was designed, namely that a single sweep has fixed at the time of sampling. For equilibrium selection with 2*Ns* = 1000 the true positive rate for SweepFinder and SweeD is between 0.32 and 0.34 (Table 5), while the true positive rate for OmegaPlus is 0.46. SweepFinder's ability to reject neutrality is improved for equilibrium selection under the SHH model when selection is strong. OmegaPlus also remains sensitive to moderate selection strengths, as the true positive rate for 2*Ns* = 100 is 0.37. Thus, LD-based approaches appear to outperform SFS-based approaches in this parameter space.

**Table 5:**
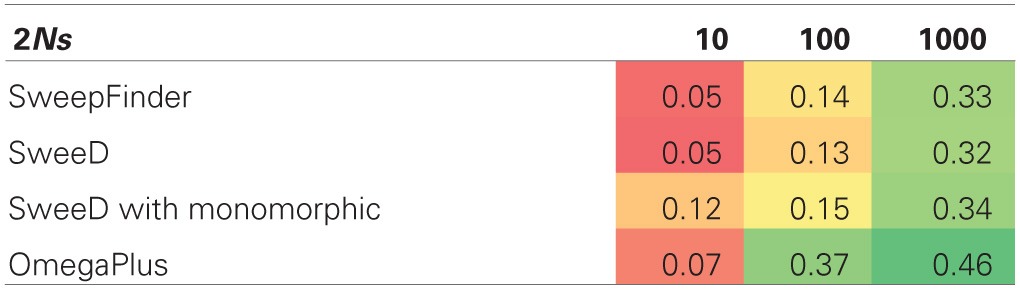
**True positive rate for SHH selection models**.

*θ = ρ = 0.001/site. Age of sweep = 0 generations for all models*.

Joint selection and bottleneck models follow a similar trend as previous models, with OmegaPlus being the only statistic with power to reject neutrality. The difference between the RHH and SHH joint models is that in RHH, the rejection rate is fairly uniform across all severities and recovery times. For the SHH models, a pattern similar to the neutral bottlenecks is observed, where the rejection rate is higher for more severe and recently recovered bottlenecks (Table 6). One reason for this uniformity when RHH is combined with various bottleneck scenarios is that multiple beneficial haplotypes are amplified during the bottleneck recovery phase. The SHH models on the other hand, experience only a single selected mutation, and thus, the underlying coalescent trees are primarily shaped by the demographic history of the population. Therefore, the demographic model determines the length of the tree, and the beneficial mutation will be at varying frequencies in the population at the recovery time, depending on the demographic model.

**Table 6:**
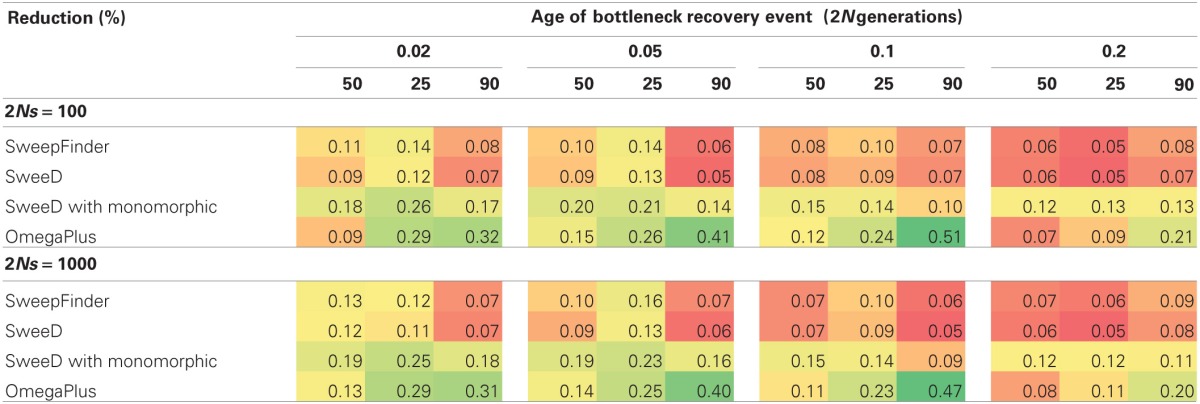
**True positive rate for joint SHH-bottleneck models**.

*θ = ρ = 0.001/site. Duration for each model is 0.25 2N generations. All bottlenecks occur at time 0 with complete recovery (back to N = 10,000) at time 0.25—age of bottleneck recovery event*.

### iHS genome-wide approach to detect significant sweeps

For iHS we initially attempted the above criteria to determine significance. However, the significance values were too large to afford any power for iHS to reject the neutral model. This may owe to the unique signal that iHS is trying to summarize, computing a score for each SNP instead of across an equally spaced grid. Extreme significant values are expected to occur in neutral haplotypes, but they appear more uniformly distributed than in a suspected sweep (Voight et al., 2006). This means that there is some requirement for extreme values to be clustered for a sweep, in order to distinguish a significant value left by a selective event from a random significant value. Thus, by binning by the number of segregating sites and using a neutral model to determine the cutoff, the extreme values of the neutral model may not be an accurate estimation of these clusters of SNPs left by a sweep.

**Figure 2:**
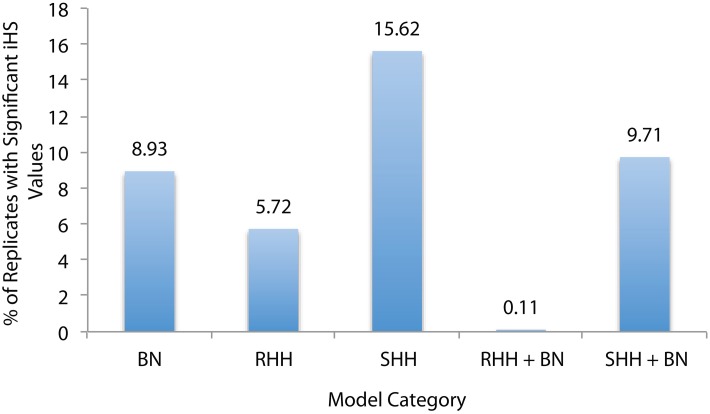
**Percentage of Sequences that Contain Selective Sweeps.** Selective sweep detection using iHS for five model categories: bottlenecks (BN), recurrent hitchihiking (RHH), single hitchhiking (SHH), and joint RHH and SHH bottleneck models. RHH models were simulated with sfcode, and SHH models were simulated with msms. These are the same models that were presented in Tables 1–6, and sequences with various selection and/or bottleneck parameters were pooled under each category. Percentages represent the number of replicates that were correctly identified as selection by iHS in each category. This plot suggests that iHS is more effective at identifying SHH events correctly, but actually many RHH replicates were eliminated due to low SNP density (see text).

For this reason we used a significance value derived from the entire dataset, following Voight et al. (2006). As they point out, this method can be useful for identifying regions of interest but does not serve as a formal significance test. To define a selective sweep signal we considered the top and bottom 1% of all iHS values as our cutoffs, and then searched for instances where iHS scores greater than or equal to these values occurred consecutively at 2 or more neighboring SNPs. In order to determine if the iHS test statistic is capable of distinguishing between a selective event and a bottleneck, we compared the fraction of sequences that contained a sweep in each model type (Figure 2).

There are two important points of note. Firstly, iHS was initially proposed as a statistic for identifying on-going sweeps—and thus, the RHH model is the most appropriate test comparison (as it contains both complete and incomplete sweeps). Secondly, iHS has a dependency on SNP dense sequences in order to be able to calculate an iHS score. For this reason, a number of replicates for 2*Ns* = 1000 were excluded from the RHH dataset because iHS was unable to calculate a value due to their low SNP density. It is also important to consider that for both SHH and RHH a majority of the sequences that appear in the outliers are from the models with weak selection (2*Ns* = 10)—again owing to the issue of SNP density. In fact, across both selection and neutral non-equilibrium models, SNP density is the main determinant of extreme iHS clusters, causing these models to appear more often in the outlier fraction. In other words, a SNP dense neutral region is more likely to contain clusters of extreme values than a less dense region that contains selective events, raising questions about the utility of iHS in looking for true selective events. For example, we observe an indirect relationship between the number of neutral bottleneck regions per model appearing in the outlier fraction, and the severity of reduction in population size, consistent with higher size reductions resulting in lower SNP density. Thus, the models within each category that appear in the outlier fraction are outliers independent of their selection model parameters.

## Summary and conclusions

For the models considered here, the SweepFinder class of statistics and iHS had the highest type II error. For both, this is likely due to their dependence on SNP density, where a higher SNP density lends more power to the statistic. Thus, the lack of power under diversity reducing models (like positive selection and population bottlenecks) led to a reduced ability to reject the neutral model regardless of the presence or absence of selection. Notably, the weakness of the Sweepfinder class of statistics is their ultimate reliance on a simulated neutral equilibrium model in order to determine significance—thus, in many ways minimizing the benefit of the “background-based” SFS notion of sweep detection as they again become model-dependent in order to calculate a *p*-value. Conversely, the weakness of the iHS class of statistics is their pure reliance on empirical outliers, thus, assuming both that positive selection has occurred in the dataset and that these selected loci will be enriched in the tails of the distribution—both of which factors account for the high proportion of neutral loci identified under bottleneck models.

OmegaPlus showed the most sensitivity to the various model parameters, with the highest true positive rates for both RHH and SHH selection, suggesting that LD-based method may be the more fruitful for detecting selective events. This statistic has difficulty distinguishing selection from a severe bottleneck however, and in RHH models with joint selection and demography, the true positive rate was uniform across all bottlenecks and within the range of true positives for equilibrium sweeps. These results emphasize the need to develop statistics that are more accurate in their identification of selective events, and are capable of dealing with these more biologically relevant models. Many natural populations are characterized by non-equilibrium histories, and the commonly used methods evaluated here are unable to deal with this effectively. However, these results also represent an important and well-quantified challenge to the field—and the performance of these statistics and the chosen parameter space can serve as a useful benchmark for future method development.

### Conflict of interest statement

The authors declare that the research was conducted in the absence of any commercial or financial relationships that could be construed as a potential conflict of interest.
